# Clinical Characteristics and Outcome of Patients With Intraoperative Aneurysm Rupture: A Retrospective Cohort Study From Nepal

**DOI:** 10.1227/neuprac.0000000000000083

**Published:** 2024-02-16

**Authors:** Mohan R. Sharma, Sandeep Bohara, Dipendra K. Shrestha, Deepak R. Joshi, Shreejana Singh, Ritesh Lamsal, Subhash P. Acharya, Prakash Kafle, Amit B. Pradhanang, Gopal Sedain, Farrokh Farrokhi, Gerald A. Grant

**Affiliations:** * Department of Neurosurgery, Tribhuvan University Teaching Hospital, Kathmandu, Nepal;; ‡ Department of Community Medicine, Institute of Medicine, Kathmandu, Nepal;; § Department of Research, Institute of Medicine, Kathmandu, Nepal;; ‖ Department of Anesthesiology, Tribhuvan University Teaching Hospital, Kathmandu, Nepal;; ¶ Department of Critical Care Medicine, Tribhuvan University Teaching Hospital, Kathmandu, Nepal;; # Department of Neurosurgery, Nobel Medical College and Teaching Hospital, Biratnagar, Nepal;; ** Department of Neurosurgery, Neuroscience Institute, Virginia Mason Franciscan Health, Seattle, Washington, USA;; ‡‡ Department of Neurosurgery, Duke University Medical Center, Durham, North Carolina, USA

**Keywords:** Aneurysm, Intraoperative rupture, Low- and middle-income countries, Microsurgery, Outcome

## Abstract

**BACKGROUND AND OBJECTIVES:**

Intraoperative aneurysm rupture (IAR) is a significant complication during microsurgical clipping of cerebral aneurysms. The timing of rupture during surgery, morphology of the aneurysm, and strategies to mitigate risk are the key factors that influence the outcome.

**METHODS:**

Consecutive patients with the diagnosis of ruptured cerebral aneurysms were retrospectively reviewed at a single University Hospital in Kathmandu, Nepal. Variables analyzed included age, sex, presenting symptoms, Hunt and Hess grades, the location of aneurysms, the timing of surgery, and intraoperative rupture status. Outcomes were assessed at 6 months after surgery.

**RESULTS:**

A total of 199 patients with 231 ruptured cerebral aneurysms from July 2014 to December 2022 were reviewed. Surgery was performed within 3 days in 60 (30.1%) patients. Twenty aneurysms ruptured intraoperatively in 20 patients (10% per patient and 8.6% per aneurysm). Patients with IAR were significantly younger (mean age 52 years) than those without IAR (mean age 58 years) (*P* < .001, 95% CI: 3.72-8.28). There was no difference in IAR rate in early vs late surgery. Anterior communicating artery complex aneurysms were noted as the most common. However, posterior inferior cerebellar and posterior cerebral artery aneurysms had the highest IAR rate, albeit with the smallest total number. Rupture during dissection was noted in 10 (50%) and during clipping in 9 (45%) procedures. Strategies for handling IAR included direct definitive clip application in 9, temporary clip-aided permanent clipping in 8, and trapping of the parent vessel in 1 patient. Although postoperative complications were significantly higher in the IAR group (*P* < .000129), the neurological outcomes using the modified Rankin scale in 6 months were similar (*P* = .877).

**CONCLUSION:**

The demographic and clinical characteristics and rates of IAR in our patient population are similar to those in the contemporary literature. In patients with IAR, the outcome is not worse than those without IAR.

**ABBREVIATIONS:**
DACA: distal anterior cerebral artery
DAMA: discharged against medical advice
DC: decompressive craniectomy
H-H: Hunt and Hess
IAR: intraoperative aneurysm rupture
LMICs: low- and middle-income countries
PCA: posterior cerebral artery
PICA: posterior inferior cerebellar artery.

The definitive treatment for aneurysmal subarachnoid hemorrhage is obliteration of the aneurysm by either microsurgical or endovascular techniques. Certain subsets of patients are best treated microsurgically based on aneurysmal characteristics or failed coiling. This is relevant in low- and middle-income countries (LMICs) because of the limited availability and affordability of endovascular therapy.^1-4^

Although the Hunt and Hess (H-H) scale and World Federation of Neurosurgical Societies grading systems offer the best prediction for surgical risks, intraoperative aneurysm rupture (IAR) is a prognostic indicator.^5,6^ The IAR has been reported to be approximately 10% (7%-35%) per patient.^7-13^ The most common risk factors for IAR are recent SAH, large aneurysms, and aneurysms of the anterior communicating artery (ACoMA) or posterior inferior cerebellar artery (PICA).^8,10,13,14^ Several studies have demonstrated a negative effect of IAR on outcomes,^11,12,15^ whereas a few have not noted such a correlation.^16^ IAR remains an unwelcome event for any neurosurgeon.

There are very few studies from LMICs regarding aneurysm rupture during surgery and its outcomes.^4,17^ In this study, we sought to describe the preoperative characteristics and outcomes of patients with IAR and compare these results with patients who did not sustain IAR.

## METHODS

Permission from the Institutional Review Committee was obtained before the data collection. Data on a consecutive cohort of patients who had undergone microsurgical clipping by a single surgeon (MRS) for cerebral aneurysms at Tribhuvan University Teaching Hospital, Kathmandu, Nepal, from July 2014 to December 2022 were retrospectively reviewed. Patients with unruptured aneurysm were excluded. Because of the retrospective nature of this study, individual informed consent was waived. A minimum of 6-month in-person follow-up was required for inclusion in the study. We followed the Strengthening the Reporting of Observational Studies in Epidemiology guideline in conducting this study.^18^

After clinical grading using the H-H scale, all patients with confirmed aneurysmal subarachnoid hemorrhage were routinely admitted to the intensive care unit under both critical care and neurosurgery teams and managed as per the departmental protocol that included the use of nimodipine, age-adjusted blood pressure targeting, and maintaining strict euvolemia. The endovascular facility in Nepal became available only in 2019, but only in private hospitals. However, the cost of treatment is beyond reach for many patients. The patients or families who chose to undergo endovascular treatment after 2019 were excluded. Patients with good grades (H-H I-III) underwent early clipping (≤3 days), whereas those in poor grades (H-H IV-V) had delayed surgery (>6 days). Intraoperative management included a craniotomy, cisternal dissection, proximal and distal artery identification, and aneurysm dissection and clipping. In the event of IAR, the anesthesiology management included brief periods of hypotension during active bleeding, hypertension (10%-20% above baseline) during temporary clipping, and maintaining a blood pressure once the aneurysm was secured. A follow-up computed tomography angiography or digital subtraction angiography was obtained in all cases for the verification of the aneurysm obliteration by 6 weeks.

For this study, we adopted the definition of IAR by Chandler et al as “bleeding that interrupts and alters the sequence of the microsurgical procedure.”^19^ We describe the following parameters: age, sex, presenting features, H-H grade, location of the aneurysm, IAR status, and management strategies. The patients were divided into two groups based on IAR status. Immediate postoperative complications and outcomes at 6 months based on the modified Rankin scale (mRS) scale^20,21^ were compared.

For descriptive statistics, the mean (± SD) was used to summarize data. The χ^2^ test was used for categorical variables and the Student's *t*-test was used for continuous variables to compare the difference in demographic, clinical, and imaging characteristics, postoperative complications, and outcomes between the two groups. *P*-values <.05 were considered statistically significant. IBM SPSS Statistics for Windows, version 26 (IBM Corp., 2019) was used for statistical analysis.

## RESULTS

During the study period, 237 cerebral aneurysms were microsurgically clipped in 204 patients. After excluding 6 patients with missing data, 199 patients with 231 aneurysms were included in the analysis. Only 6 patients of 38 from 2019 to 2022 underwent endovascular treatment. These 38 patients were part of the 199 cohorts. The COVID-19 pandemic contributed to the lower number of cases during this period. Thirty patients (15.1%) had multiple aneurysms. Twenty aneurysms ruptured intraoperatively in 20 patients (10% per patient and 8.6% per aneurysm).

### Baseline Characteristics

Table 1 shows the baseline characteristics of patients with and without IAR. The age ranged from 17 to 83 years. There was a significant difference in the mean age between the two groups (IAR: 52 years, non-IAR: 58 years; *P* = .001, 95% CI: 3.72-8.28). There was a strong sex predilection in both groups; the difference, however, was not statistically significant.

**TABLE 1.** Baseline Characteristics of the Patients With and Without Intraoperative Rupture (n = 199)

Parameters	IAR	Non-IAR	*P* value^a^
Age in years (mean)	52 (±4)	58 (±5)	.001 (CI: 3.72-8.28)
Sex ratio (M:F)	8:12 (1:1.5)	40:139 (1:3.5)	.077
Smoking	15 (75%)	104 (57.8%)	.770
Hypertension	10 (50%)	96 (543.3%)	.990

IAR, intraoperative aneurysm rupture.

a Two-tailed *P* value.

### Clinical Features

The clinical features at presentation are shown in Figure; headache was present in all patients. The admission H-H scale is shown in Table 2. Most patients in both groups were in good grades at presentation. On dichotomizing those with good (I-III) and poor grades (IV-V), there was no statistical difference between the two groups (*P* = .694).

**FIGURE.**
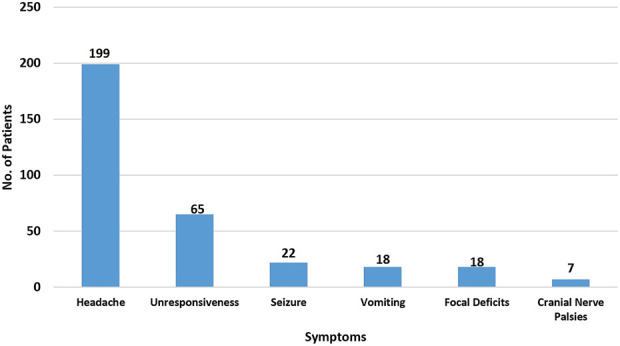
Bar graph showing the frequency of clinical features in 199 patients.

**TABLE 2.** H-H Grade of Patients With (20) and Without (179) IAR (n = 199)

H-H grade	IAR	Non-IAR	*P* value
I	2 (10%)	30 (16.8%)	.694
II	10 (50)	110 (61.5%)	
III	6 (30%)	21 (11.7%)	
IV	1 (5%)	14 (7.8%)	
V	1 (5%)	4 (2.2)	

H-H, Hunt and Hess; IAR, intraoperative aneurysm rupture.

One hundred twenty patients (60.0%) presented within 3 days, 64 (32.2%) between 3 and 10 days, and 15 (7.5%) after 10 days of ictus. Sixty (30.1%) patients were operated within 3 days. There was no correlation between the day of surgery after ictus and the risk of IAR (Table 3).

**TABLE 3.** Days of Ictus and Surgery, and the Risk of Intraoperative Rupture in Patients With (20) and Without (179) IAR (n = 199)

Parameters	IAR	Non-IAR	*P* value
Day of ictus to presentation ≤3 days	15 (75%)	127 (70.9%)	.982
Day of ictus to surgery ≤3 days	10 (50%)	50 (27.9%)	.243

IAR, intraoperative aneurysm rupture.

### Aneurysm Characteristics

A total of 209 aneurysms including those in the posterior communicating artery (PCoMA) were in the anterior circulation and 18 in the posterior circulation. Two patients had both anterior and posterior circulation aneurysms as part of multiple aneurysms. A total of 17 anterior and 3 posterior circulation aneurysms bled intraoperatively (Table 4). The aneurysms in the ACoMA (8) followed by the middle cerebral artery (MCA) (4) were prone to IAR. However, the IAR rate was highest for PICA aneurysms.

**TABLE 4.** Distribution of Aneurysms With and Without Intraoperative Rupture (n = 231)

Distribution of aneurysms	IAR	Non-IAR	IAR rate (%)
ACoMA	8	64	11.1
MCA	4	50	7.4
PCoMA	2	34	5.6
ICA	1	27	3.6
DACA	2	19	9.5
PICA	2	6	25.0
PCA	1	6	14.3
Basilar tip	0	5	0

ACoMA, anterior communicating artery; DACA, distal anterior cerebral artery; IAR, intraoperative aneurysm rupture; ICA, internal carotid artery; MCA, middle cerebral artery; PCA, posterior cerebral artery; PCoMA, posterior communicating artery; PICA, posterior inferior cerebellar artery.

ICA aneurysms include those arising from the clinoid, ophthalmic, superior hypophyseal, and bifurcation segments.

Eleven patients had giant (size >25 mm) aneurysms: 2 in the IAR group and 9 in the non-IAR group.

### Phases of Dissection and Rupture

One aneurysm ruptured before visualization, 10 during dissection, and 9 during clip placement. Rupture during clip application was attributed to incomplete dissection in 5, poor clip application in 4, and clip applicator failure in 1 procedure.

### Technical Strategies for Handling the Rupture

In the patient with rupture before visualizing the aneurysm, the use of two wide-bore suctions, direct pressure with cottonoid patties at the presumed site of the aneurysm, rapid hematoma evacuation, identification of aneurysm, and clipping were performed. A direct permanent clip was placed in 9 patients, whereas in 8, temporary clipped–aided permanent clipping was performed. In 2 patients, pilot clips were placed at the rupture site before definitive ligation. Trapping was performed in a patient with connective tissue disorder with a large right saccular internal carotid artery (ICA) aneurysm. The right ICA was transected during the procedure. However, there was no deficit postoperatively.

### Postoperative Complications

As shown in Table 5, postoperative complications were documented in 34 (17.1%) patients—10 in the IAR group and 24 in the non-IAR group (*P* < .001; 95% CI: 15.72%-57.11%). Subgroup analysis was not possible because of the small sample size. Contralateral blindness occurred in 1 patient with an ACoMA aneurysm that ruptured during surgery. Both paraplegia and diabetes insipidus occurred in patients with ACoMA aneurysms. One patient with a left vertebro-PICA aneurysm required a permanent feeding jejunostomy.

**TABLE 5.** Postoperative Complications in Patients With (20) and Without (179) IAR (n = 199)

Complications	IAR	Non-IAR
Postoperative brain swelling requiring decompression	10 (50%)	18 (10%)
Ventilator-associated pneumonia	8 (40%)	10 (5.6%)
Cranial nerve palsies	6 (30%)	13 (7.3%)
Hydrocephalus requiring shunt	6 (30%)	10 (5.6%)
Paraplegia	2 (20%)	0
Diabetes insipidus	2 (20%)	0
Permanent feeding jejunostomy	1 (10%)	0
Contralateral blindness	1 (10)	0

IAR, intraoperative aneurysm rupture.

### Outcome

The follow-up period ranged from 6 months to 96 months (mean 58 months). Three patients (2 with IAR and 1 without IAR) were discharged against medical advice and are categorized under unfavorable outcomes. Altogether, 155 (77.89%) patients had a favorable outcome (mRS 0-2) in 6 months (Table 6). A total of 18 patients (3 in the IAR group and 15 in the non-IAR group) died in our series, leading to a mortality of 9.1%. The difference in unfavorable outcomes was, however, not statistically significant.

**TABLE 6.** The Outcome of Patients With and Without IAR (n = 199)

Outcomes	IAR	Non-IAR	*P* value
Good (mRS 0-2)	14 (70.0%)	140 (78.3%)	.877
Poor (mRS 3-6)	6 (30.0%; 3 deaths, 2 DAMA, and 1 dependent)	39 (21.5%; 15 deaths, 1 DAMA, and 23 dependent)	

DAMA, discharged against medical advice; IAR, intraoperative aneurysm rupture; mRS, modified Rankin scale.

## DISCUSSION

This is a contemporary series of patients with microsurgically treated cerebral aneurysms and their outcomes based on IAR status from a single university hospital in Nepal. Although small from a global viewpoint, this is a relatively large series from LMICs and serves to provide a unique perspective on management. The resource limitations (availability of operation time, dedicated neuroanesthesia, and neurocritical care team) typical of LMICs in the diagnosis and timely treatment of this complex disease are the most likely cause of the low case volume in our series. In recent years, the impact of the availability of dedicated neuroanesthesia and critical care teams and the regionalization of neurosurgical care are beginning to be realized.

Successful management of IAR involves many factors: the location of aneurysms, the stage of dissection, the experience of the treating surgeon, availability of a high-end microscope, and if needed, capability to perform bypass procedures.^8,11,13^ Working on a “slack brain” under high magnification and sharp dissection are known to decrease the rate of premature rupture.^7,8,11,13,22^ The expertise of a neuro-anesthesiologist during surgery cannot be overemphasized. All these may not be readily available in all centers in LMICs and can make the management of IAR uniquely difficult.

### Rate of Intraoperative Aneurysm Rupture

In our series, the IAR was 10% per patient and 8.6% per aneurysm. These figures are consistent with previously published reports. In a large systematic review performed in 2021, of all intraoperative surgical adverse events, the IAR was the most common (median rate of 16.6%).^16^

Another review in 2013 consisting of 9488 patients reported the incidence as 9.2% per aneurysm.^23^ Leipzig et al^8^ in a series of 1694 saccular aneurysms noted IAR rates of 6.7% per aneurysm and 8.9% per patient. A recent study from India reported the IAR rate as 16.4% per aneurysm, whereas in a previous study from Nepal, the rate was 12.1%.^4,17^ The wide variation of IAR is partly attributed to the different operational definitions of the IAR, heterogeneous patient populations, and variable experience of the operating surgeons.

### Demographic Characteristics and Grading

The demographic characteristics of our patients were similar to those observed in other series.^4,7,8,17,24^ The only variable with a statistical difference was the mean age of the patients, with a slightly younger population that suffered an IAR. Contrary to our findings, increasing age was found to be a risk factor in a study by Swiatnicki et al.^25^ There is a stronger female sex predilection in our series, which corroborates with previously published studies and is not a risk factor for IAR.^4,7,8,24^

The H-H grades were comparable in both groups in this study, which corroborates with previous studies.^8,14,17,24,26^ However, in a series of 903 patients by Oppong et al, the severity of SAH was a predictor of IAR.^24^ By contrast, Radhakrishna et al in their series documented more IAR in good-grade patients.^17^

The high rate of good-grade patients in our setting may be explained by poor-grade patients not reaching the hospital because of remote locations and difficulty in transfer.

### Characteristics of Aneurysms

The PICA aneurysms had the highest rate of rupture followed by posterior cerebral artery and ACoMA aneurysms. Among all sites, ACoMA, PCoMA, MCA, and PICA aneurysms demonstrate a higher incidence of rupture.^8,24,27^ Why aneurysms of certain locations are more prone to rupture remains open to speculation. Working in a difficult area of the brain and the fragile nature of aneurysms in certain locations are potential contributors. In PCoMA and MCA locations, early retraction may lead to premature rupture.^10,13^ In patients with large hematomas, rupture can follow a sudden decompression causing pressure differential on the aneurysm sac.^10^ In ACoMA aneurysms, the same morphological or hemodynamic parameters that make these aneurysms the most frequent sites of first rupture may play a role during surgery also.^28^

Our limited data sets did not allow size differentiation other than giant vs nongiant. A total of 11 patients (6.2%) had giant aneurysms, of which only 2 patients suffered IAR. Large aneurysms are known to be more prone to rupture.^24^ However, in the study by Inci and Karakaya, the size of the aneurysm was not a risk factor for rupture.^7^

### Timing of Surgery and Rupture

Our data showed no difference in the IAR rate between the early vs Late surgery groups. This again could be because of the small sample size. Early surgery is reported to be associated with an increased risk of IAR by some studies,^14,27^ whereas others have found no such difference.^4,8,14,17,29^ The increased risk with early surgery is arguably because of the dislodgement of immature clot sealing the rupture site and technical difficulties in dissection in a relatively tight brain because of recent SAH.^29,30^

### Stage of Dissection and Rupture

Most ruptures occur during either aneurysm dissection or clip application.^8,11,19,31-34^ In our series, the IAR was noted during either dissection or clip application in all but 1 patient. Sharp dissection is associated with a lower risk of rupture compared with blunt dissection.^11,13^ However, these data were not available for analysis in our study.

### Management of Rupture

The management of IAR largely depends on the stage of dissection. Volume–outcome relationships have been the topics of extensive discussion regarding neurovascular surgery, especially for aneurysm treatment. In those aneurysms where the neck has already been dissected, direct clip placement is recommended. This was performed in 8 instances in our series. In situations when the aneurysm ruptures at the dome prematurely, pilot clip placements allow further dissection of the aneurysm and permanent clip application. Temporary clips are reserved in the early part of dissection. However, the use of temporary clips can be associated with postoperative stroke to a variable degree and their safest duration is still controversial.^35-38^ Our sample size is too small to analyze this association.

### Postoperative Complications and Outcome

The most common postoperative complication was brain swelling requiring a decompressive craniectomy (DC) followed by pneumonia. This 14% rate of DC is consistent with the previously published literature varying from 3.3% to 25.6%.^39^ There was a significant difference in the rate of postoperative complications between the two groups. More patients requiring DC in the rupture group suggested that the attempts to manage the IAR probably led to increased brain injury because of tissue handling, prolonged brain retraction, hemodynamic instability, and the use of temporary clips.^11,17^

A total of 77.5% of our patients had favorable outcomes (mRS 0-2) contrary to a large multicentric series from Brazil that noted favorable outcomes in about 60% of cases.^40^ More patients being in good grades and operation by a single surgeon could explain the more favorable outcomes in our series. There was no difference in outcome between those with and without IAR. In the series of 305 patients by Batjer and Samson, the outcomes were significantly worse than in those with IAR.^11^

Although we had more complications in the IAR group, there was no correlation with the outcome at 6 months, similar to the results by Kassell et al.^29,41^ This nonsignificant correlation of complications with outcomes could be because of multiple reasons: (a) this is a low powered study with selection bias, (b) more IARs occurred during the final stage of dissection where direct clip application was possible, and (c) increasing experience of the surgeon. The start of a neurosurgery residency program in 2010, the availability of specialty-trained neuroanesthetists from 2019, and the establishment of a dedicated neurocritical care team in 2020 are the main drivers of the improved care.

The 9.0% mortality in our series (15% in the IAR group and 8.3% in the non-IAR group) is comparable with that reported in the literature from different parts of the world, which varies from 6.6% to 36%.^7,8,13,17,24,27,42^

### Limitations

This study is a retrospective, single-institutional study on patients treated by a single surgeon. This limits the wider generalizability of results. Why our patients were relatively younger and fared better regarding outcomes could be because those who were very sick or elderly could not make it to the hospital, which introduces a selection bias. We did not grade the severity of IAR, which might have a potential impact on the outcome. We acknowledge that the sample size is inadequate for subgroup analysis. In addition, we could not present our results based on the aneurysm size, duration of temporary clipping, and the presence of atherosclerotic changes in the aneurysm because of inadequate information in our database.

## CONCLUSION

The PICA, PCoMA, and ACoMA aneurysms have higher IAR rates compared with aneurysms at other locations. Although there were no preoperative risk factors for rupture during surgery except for age, it was associated with increased postoperative complications but a similar 6-month outcome. Further large multicentric studies in LMICs are strongly recommended to better define the risk factors for IAR and their effects on neurological outcomes.
